# Predicting auditory space calibration from recent multisensory experience

**DOI:** 10.1007/s00221-015-4259-z

**Published:** 2015-03-21

**Authors:** Catarina Mendonça, Andreas Escher, Steven van de Par, Hans Colonius

**Affiliations:** Department of Signal Processing and Acoustics, Aalto University, Otakaari 5, 02150 Espoo, Finland; Department of Psychology, Cluster of Excellence Hearing4all, Carl von Ossietzky University, Oldenburg, Germany; Department of Medical Physics and Acoustics, Cluster of Excellence Hearing4all, Carl von Ossietzky University, Oldenburg, Germany

**Keywords:** Audiovisual, Localization, Shift, Adaptation, Ventriloquism, Ventriloquism aftereffect

## Abstract

Multisensory experience can lead to auditory space recalibration. After exposure to discrepant audiovisual stimulation, sound percepts are displaced in space, in the direction of the previous visual stimulation. This study focuses on identifying the factors in recent sensory experience leading to such auditory space shifts. Sequences of five audiovisual pairs were presented, each randomly congruent or discrepant in space. Each sequence was followed by a single auditory trial and two visual trials. In each trial, participants had to identify the perceived stimuli positions. We found that auditory localization is shifted during audiovisual discrepant trials and during subsequent auditory trials, suggesting a recalibration effect. Time did not lead to greater recalibration effects. The last audiovisual trial affects the subsequent auditory shift the most. The number of discrepant trials in a sequence, and the number of consecutive trials in sequence, also correlated with the subsequent auditory shift. To estimate the individual contribution of previously presented trials to the recalibration effect, a best-fitting model was developed to predict the shift in a linear weighted combination of stimulus features: (1) whether matching or discrepant trials occurred in the sequence, (2) total number of discrepant trials, and (3) maximum number of consecutive discrepant trials, (4) whether the last trial was discrepant or not. The selected model consists of a function including as properties the type of stimulus of the last audiovisual sequence trial and the overall probability of mismatching trials in sequence.

## Introduction

Auditory space is not represented directly in the sounds that reach the ears. It must be inferred from a combination of interaural differences and spectral cues (e.g., Ahveninen et al. 2014; Middlebrooks and Green 1991). The auditory cues are processed and lead to a perceived sound space. But throughout life, auditory localization cues vary, mostly due to anatomical or hearing sensitivity changes, requiring successive new cue-to-space associations (for a recent review, see Mendonça 2014). It is nowadays well accepted that auditory space is continuously calibrated through sensory experience (e.g., King et al. 2001). Multisensory experience is thought to play a major role in such calibration processes, with great emphasis on audiovisual interactions. Since the visual space has a direct correspondence to the visual receptive field, there is great accuracy, and therefore reliability, in localization from vision. It is therefore not surprising that, in audiovisual localization, vision outweighs audition. In a phenomenon known as visual capture or ventriloquism, when visual and auditory sources are presented in discrepant spatial positions, the sound is often perceived as overlapping the visual source, or at least closer to it (Pick et al. 1969; Alais and Burr 2004; Chen and Vroomen 2013). More importantly, when humans are continuously exposed to such discrepant audiovisual stimulations, the subsequent unisensory auditory localization is also changed, and auditory-only percepts are shifted in the same direction as during the audiovisual stimulation. This phenomenon is referred to as the ventriloquism aftereffect (Radeau and Bertelson 1974; Recanzone 1998).

Most studies on the ventriloquism aftereffect induced shifts in auditory localization by exposing the subjects to consecutive discrepant audiovisual pairs, over several blocks or one long adaptation block (e.g., Recanzone 1998, Lewald 2002; Frissen et al. 2003; Radeau and Bertelson 1974; Kopčo et al. 2009; Wozny and Shams 2011a). The onset and duration of this effect are not yet clearly understood. Frissen et al. (2012) have identified that aftereffects occurred after only 18–24 exposures to audiovisual discrepant stimulation. However, it has recently been shown that, after a single audiovisual discrepant trial, a subsequent auditory shift can already be observed. Wozny and Shams (2011b) presented visual, audiovisual, and auditory trials in random order, with the audiovisual trials varying in spatial discrepancy from −26° to 26°. They found that, after audiovisual discrepant trials, subjects showed a localization shift in the subsequent auditory trial. The larger the discrepancy in the audiovisual trial, the larger the shift in the auditory trial. The authors reported further evidence that an auditory shift could still be found when the discrepant trial occurred two or three trials prior to the auditory trial. A trend to shift auditory localization was observed when the discrepant trials occurred up to five trials before the auditory trial.

In this study, we were interested in further exploring how recent audiovisual stimulation leads to auditory space shifts. We intended to replicate the findings by Wozny and Shams (2011b) and to quantify the relevance of each of the five audiovisual trials prior to the auditory localization estimate. Wozny and Shams (2011b) did not design their experiments specifically to analyze sequential effects. The main hypothesis in this study was that the most recent audiovisual stimulus would affect the subsequent auditory localization, but other recent stimulation could also affect it. It was hypothesized that the magnitude of such effect could also depend on factors such as number of consecutive discrepant trials, or total number of discrepant trials in recent sensory experience. Therefore, our main goals were to analyze the sequence of multisensory events leading to the auditory shifts and to identify the factors predicting such shifts. For that purpose, we devised an experiment with sequences of five audiovisual events randomly selected to be either matching or discrepant in space, each time followed by a single auditory trial. Data analysis focused on relating the type of multisensory sequence to the size of the auditory localization aftereffects.

## Methods

### Participants

Eleven subjects, aged 19–31, took part in the experiment. Three participants were female, and one male was author. Subjects were paid for their participation. All subjects provided a signed informed consent. The experiment was conducted in accordance with the ethical standards described in the 1964 Declaration of Helsinki. All subjects had normal or corrected-to-normal vision, and none reported any known hearing deficits.

### Apparatus and Stimuli

Experiments were conducted in a large anechoic chamber, in total darkness. There was a semicircular array of speakers and LED lights spanning from −40 to +40 degree in azimuth, which were all used for data collection. However, the visual stimuli were only presented within ±20°. There was one red LED light per degree and one additional yellow LED light at azimuth 0. There were 18 speakers, ranging from −42.2° to +42.2°. The auditory stimuli were presented within ∓32°. Using amplitude and phase panning, it was possible to render sounds at any position between speakers.

There were auditory, visual and audiovisual stimuli. Auditory stimuli were Gaussian white noise bursts, at 60 dB SPL, lasting for 350 ms. Visual stimuli were red light flashes at 51 cd/m^2^ lasting for 350 ms. Audiovisual trials displayed simultaneously a flash and a noise burst. There were 3 stimulus areas defined. Within these areas, stimulus positions were randomly selected in all trials: from −5° to +5°; from −20 to −10°; from +10 to +20°. There were two types of audiovisual trials: matching and discrepant. In *matching* trials, sound and light positions were the same. In *discrepant* trials, sounds were always shifted by 12°. The magnitude of the shift was determined in pretests, where we compared several shifts (5°–15°) and identified a discrepancy at which subsequent shifts could be observed in all participants, after most sequences.

Responses were collected for all trials within a sequence through a slider with which subjects could finely choose the perceived stimulus position in the array. By using the slider, the LEDs in the whole array (∓40°) were activated. The position of the active LED could be adjusted until it matched the position the subject wanted to report, after which subjects clicked a button to log the response. After unisensory trials, only one estimate was reported. After audiovisual trials, subjects provided two estimates to log the response, first for the light and then the sound. It was determined in a pretest that the order in which the response to each sensory modality was presented did not affect the results. Subjects were instructed that, if stimuli were perceived as co-localized, they could simply select the desired LED and press twice.

Half of the participants undertook the experiment with sounds consistently shifted to the right, and the other half of the subjects had the sounds shifted to the left. The choice of having all-discrepant trials with the same relation between visual and auditory stimulus had to do with two factors: first, because up to the date of the implementation of this experiment, all studies but one had analyzed the ventriloquism aftereffect after prolonged exposure to audiovisual pairs that were always congruently discrepant; and, second, because the one study that presented random audiovisual pairs (Wozny and Shams 2011a, b) experienced methodological limitations. Indeed, that study used a total of 146 subjects, a very uncommon sample size in perception studies. It can be assumed that such paradigm might raise the response variability within and across participants. In our study, to collect a single auditory localization estimate, we had to present a sequence of 8 trials and collected a total of 13 responses (see next section). This made the experiment much longer than previous experiments on the topic. It would therefore be unpractical to multiply the types of sequence and number of repetitions by presenting varying directions of discrepant stimuli.

### Procedure

Before each stimulus presentation, a yellow light was displayed at azimuth 0° for 0.8 s. Subjects were asked to align nose and eyes with the yellow light. Head position was stabilized through a headrest. Subjects had to avoid any head movements. Eye movements were not controlled for, since they have been shown to have no effect on the ventriloquism effect (Bertelson et al. 2000; van Barneveld and van Wanrooij 2013). The time interval between the fixation light and stimulus onset varied randomly between 0.5 and 1 s.

**Fig. 1:**
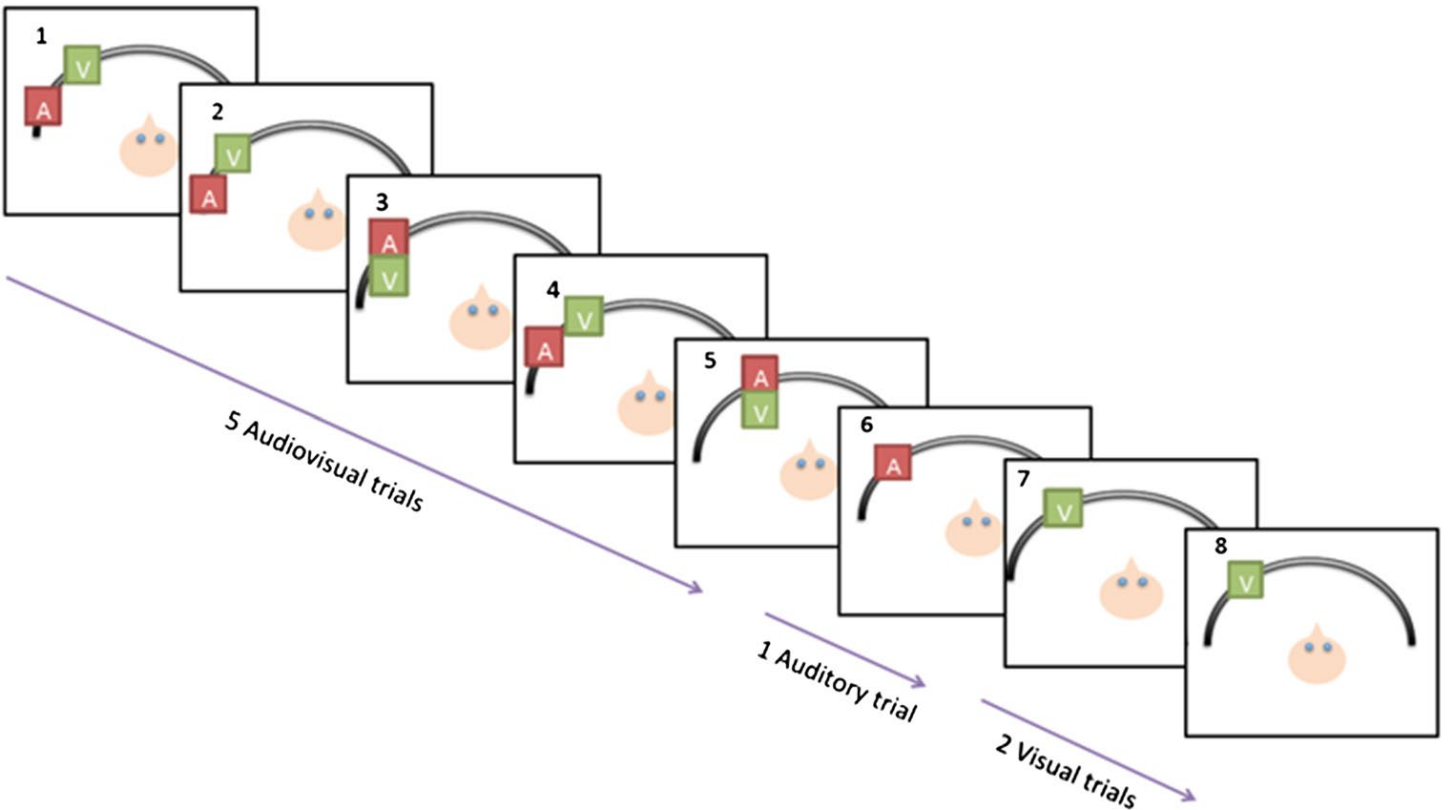
Example of one sequence of trials. All sequences started with five audiovisual trials (*1*–*5*) and were followed by one auditory trial (*6*) and two visual trials (*7* and *8*). In the example, the audiovisual sequence started with a discrepant trial (*1*) and ended with a matching trial (*5*). For this subject, all-discrepant trials had the auditory stimulus 12**°** to the *left* of the visual stimulus. In this sequence, stimuli were presented in the *left* stimulus area

The stimuli were presented as sequences of different trial types. Each sequence started with five audiovisual trials and was followed by an auditory and two visual trials (Fig. 1 shows one example sequence). Each sequence had all stimuli within only one of the three stimulus areas. The sequences of five audiovisual trials had a random combination of matching and discrepant trials, and therefore the total number of possible sequence types was 32.

The two final visual trials served to create a gap between sequences and also served to obtain the distribution of the visual-only localization estimates.

Subjects performed exactly nine repetitions per sequence type. The experiment took approximately 3 h per subject. Subjects could take a break whenever desired. If no breaks were requested, a mandatory break would occur every 30 min. Subjects broke down the experiment into as many days as needed to complete the experiment. Before data collection begun, there was a familiarization block lasting for 5 min, during which no feedback was provided.

## Results

The results are organized into three subsections. First, general localization results are presented with a focus on auditory localization shifts independent of prior presented stimuli. Then, auditory localization shifts are analyzed as a function of previous stimuli. Finally, a simple model predicting the auditory localization shifts based on prior presented stimulus type is proposed.

### Localization

**Fig. 2:**
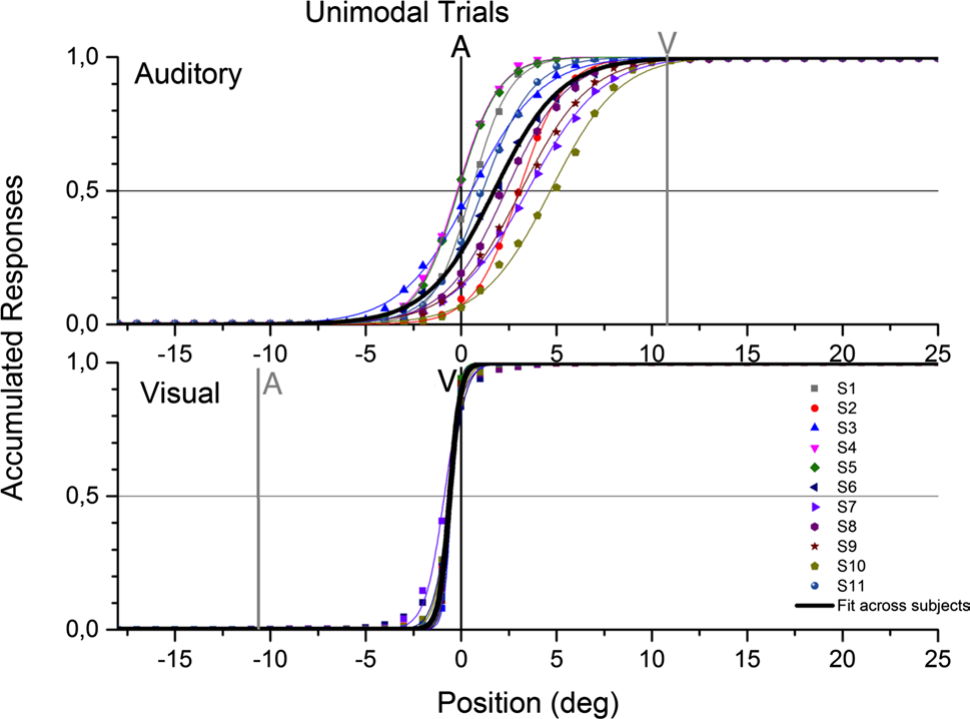
Cumulative responses in unimodal trials. The scale has been collapsed so that 0° corresponds to stimulus position. In the auditory subplot, the position of the visual stimulus in the previous audiovisual discrepant trials was at +12° (*gray line*). In the visual subplot, the position of the auditory stimulus in the previous audiovisual discrepant trials was at −12° (*gray line*). Data points correspond to the cumulative proportion of responses at each spatial position relative to the source. For example, the values at 5° reveal the proportion of times the stimuli were localized anywhere in the *left* and up to 5° to the *right* of the stimulus. Data points are per subject (S1–S11), and each represents 288 trials in the auditory subplot and 576 trials in the visual subplot. The scale has been obtained by subtracting the stimulus position to the stimulus response, correcting across subjects with different orientation of discrepant trials, and computing the cumulative relative frequencies. The psychometric curves were obtained using the logistic function. Auditory estimates were shifted and varied across participants. Visual estimates were accurate and stable across participants (color figure online)

In this section, data are analyzed without regarding the prior stimulus type that was presented. There were no differences between localization of visual and auditory stimuli presented in the central, left, or right area, in the bimodal and unimodal trials. Therefore, all data are presented together. In Fig. 2, localization results are shown for auditory and visual trials. In that figure, responses were normalized for each subject by subtracting all pooled stimulus responses to the corresponding stimulus positions. Note that half of the subjects always had the auditory stimulus to the left of the visual stimulus and the other half were presented in the reverse way. In the later case, data were inversed such that results can be collapsed across the two groups.

On average, all subjects localized the visual source close to its position, but presented a small shift in the perceived auditory source locations. The individual and across-subject localization results of the unimodal trials are presented in Fig. 2.

The visual responses were very accurate, with a mean localization position at −0.067° (SD = 0.73°). Please note that in Fig. 2, the visual psychometric function appears slightly shifted to the left due to an effect of the cumulative function: since most responses are precisely at 0°, the values at that point are closer to 1 and therefore are not at 0.5 as in traditional psychometric distributions from two-alternative forced choice methods. The distribution of the visual responses was not different from the normal distribution in a Kolmogorov–Smirnov test (*D*_10_ = 0.206, *p* = 0.2). In a *t* test, it was found that the unimodal visual localization responses did not differ from the true stimulus position (*t*_10_ = 0.3044, *p* = 0.767). The auditory responses were shifted toward the position of the visual stimulus in the previous audiovisual discrepant trials by an average of 2.2° (SD = 2.52°). Data from unimodal auditory data were not different from the normal distribution in a Kolmogorov–Smirnov test (*D*_10_ = 0.155, *p* = 0.2). In a *t* test, it was found that the auditory localization shift, tested as the two-tailed difference between the response distribution and zero, was statistically significant (*t*_10_ = 2.8932, *p* < 0.05).

It must be noted that the auditory estimates plotted in Fig. 2 are averages of all estimates, obtained after all types of sequences. Since all-discrepant trials presented an audiovisual mismatch in the same direction, subjects were exposed to an average of 6° mismatch. This could lead to a recalibration mechanism, by which subjects accumulate evidence regarding average audiovisual mismatches and adapt their estimates accordingly. It was found that the average auditory shift after a sequence of all-matching trials was 1.48°, while the average shift after a sequence of all-discrepant trials was 3.48°. The results from the all-matching sequences show that, indeed, despite recent sensory experience correcting the auditory shifts, a background, possibly longer-term, recalibration effect took place, due to the prolonged exposure to audiovisual pairs that were, on average, shifted. The results from the all-discrepant sequences show that, despite such longer-term recalibration, recent sensory experience greatly impacts the localization shift

By considering only the audiovisual discrepant trials, the results also allow us to assess the extent of the immediate ventriloquist effect. The results obtained during the audiovisual discrepant trials were similar to those obtained in the unimodal trials (Table 1). The visual responses were quite accurately localized (mean = −0.09°), but there was a higher response variability (SD = 1.51°). The response distribution did not differ from normality (*D*_10_ = 0.203, *p* = 0.2). It was found that the visual localization responses did not differ from the stimulus position in the discrepant trials (*t*_10_ = 0.1908 *p* = 0.847). The auditory responses were shifted in the direction of the light stimulus by an average of 4.16° (SD = 2.88°). Response distribution was not different from the normal distribution (*D*_10_ = 0.208, *p* = 0.2). The auditory shift was statistically significant (*t*_10_ = 4.7973, *p* < 0.001), revealing a multi-sensory bias on the auditory localization due to the presence of the visual stimulus. In audiovisual discrepant trials, visual and auditory stimuli were only rarely reported as co-localized. More specifically, only in 5 % of the trials did subjects provide identical visual and auditory estimates. However, there was great individual variability at this level. Two subjects never reported the stimuli as co-localized, while one subject did so in as many as 39 % of the trials. Therefore, it seems that subjects mostly did not perceive the audiovisual discrepant events as a unitary event. This would mean that the calibration effects found in the subsequent auditory trials cannot be explained by perceived unity during the audiovisual experience.

**Table 1:** Summary of localization mean and standard deviation (SD) in unimodal, audiovisual discrepant, and audiovisual matching trials

Condition	Mean visual	SD visual	Mean auditory	SD auditory
Unimodal	0.067°	0.73°	2.2°	2.52°
Discrepant	−0.09°	1.51°	4.16°	2.88°
Matching	0.07°	0.94°	1.28°	2.18°

In the audiovisual matching trials, the visual stimulus was once again well localized (mean 0.07°). Response variability (SD = 0.94°) was higher than in the unimodal visual trials, but lower than in audiovisual discrepant trials. The auditory stimulus in the matching trials was still perceived as slightly shifted (mean 1.28°), although less so than in the auditory and audiovisual discrepant trials. Response variability was also large (SD = 2.18°), but less than in the remaining auditory conditions. In matching trials, both the visual and auditory response distributions were not different from the normal distribution (visual *D*_10_ = 0.173, *p* = 0.2; auditory *D*_10_ = 0.231, *p* = 0.1). In *t* tests for the audiovisual matching trials, it was found that the visual localization responses did not differ from the stimulus position (*t*_10_ = 0.2327, *p* = 0.821), and neither did the auditory responses (*t*_10_ = 1.9474, *p* = 0.08), although there was a trend toward significance. In an average of 47 % of the trials, visual and auditory stimuli were reported as being at exactly the same location.

### Auditory shifts

So far, results presented all data pooled together without distinguishing between prior presented trials. In this section, we take a look into auditory localization shifts, accounting for time and for the events in the sequence. The auditory shift was calculated as the difference between the auditory stimulus position and the auditory position estimate, as given by:

1 $${\text{SH}}_{\text{A}} = \hat{S}_{\text{A}} - S_{\text{A}}$$

where ${\text{SH}}_{\text{A}}$ is the auditory shift, $S_{\text{A}}$ is the auditory stimulus position and $\hat{S}_{\text{A}}$ is the auditory position subjective response. This formula was applied to data from subjects that had light to the right of the auditory stimuli; the remaining subjects had their results further multiplied by −1.

**Fig. 3:**
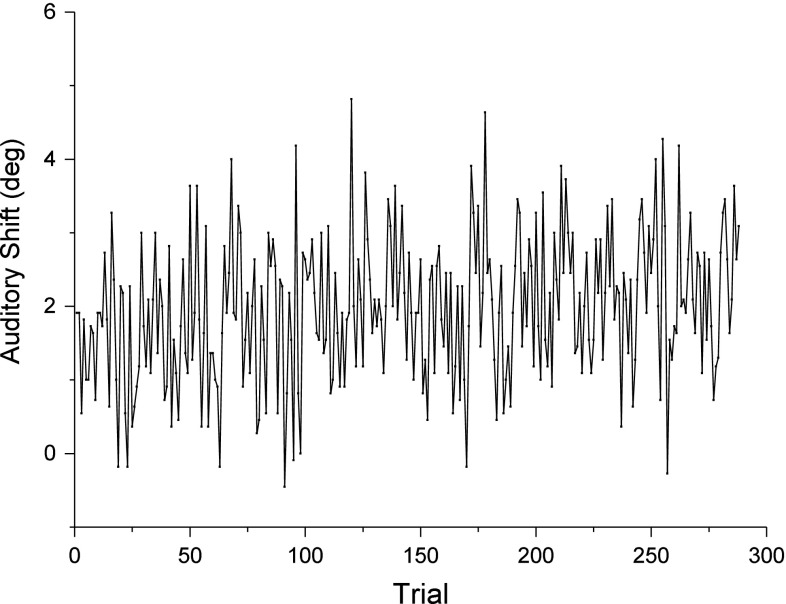
Average auditory localization shift as a function of experiment trial. There were a total of 288 auditory trials in the experiment. Each data point corresponds to an average across 11 subjects for each of the 288 trials

In Fig. 3, the average auditory shift is presented as a function of auditory trial number. Since each auditory trial happened after a sequence of audiovisual stimuli, higher trial numbers mean more accumulated exposure to discrepant and matching stimuli.

From Fig. 3, it becomes visible that there was no clear trend associated with time. To better test for the time effect, trials were grouped into 4 blocks of 72 trials each. The average shift in the first block was 1.95°, followed by 2.05°, 2.19°, and 1.96° in the second, third, and fourth blocks, respectively. In a one-way ANOVA, it was found that the shift did not vary across blocks [*F*(3) = 0.287, *p* = 0.834].

**Fig. 4:**
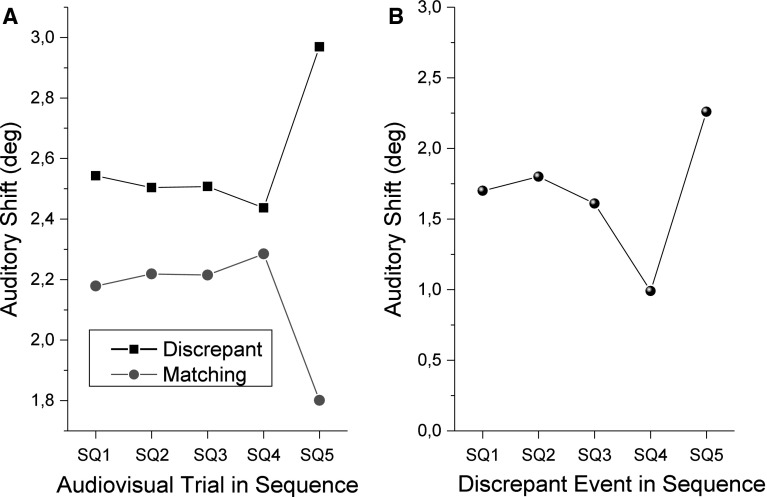
Localization shifts in auditory trials as a function of previous sequence. A Shifts when audiovisual sequence trials 1 (SQ1) to 5 (SQ5) were discrepant (*black*) or matching (*red*). B Shifts in the case of sequences with only one discrepant trial and four matching trials. Values show the shift when only the first (SQ1) to fifth (SQ5) trials were discrepant (color figure online)

To analyze in more detail the localization shifts in the auditory trials due to prior presented sequences, we calculated the average response shift observed when each of the five audiovisual trials (SQ1–SQ5) was discrepant or not (Fig. 4a). As an example, in Fig. 4a, the leftmost red (circle) value reports the average shift in auditory localization after all sequences in which the first event of the sequence (SQ1) was matching.

It was found that when any of the trials in the sequence was discrepant, there was a larger shift in the subsequent auditory trial. Shifts were of 2.54°, 2.5°, 2.51°, 2.44°, and 2.97° when trial SQ1, SQ2, SQ3, SQ4, and SQ5 were discrepant, respectively. Conversely, shifts were of 2.18°, 2.2°, 2.21°, 2.28°, and 1.8° when trial SQ1, SQ2, SQ3, SQ4, and SQ5 were matching, respectively. According to these values, the sequence trial which led to greater changes in shift was the last one in the sequence, while the fourth trial had the smallest impact on the subsequent auditory shift.

When all but one event in the sequence were matching (Fig. 4b), a similar effect was observed. There was a greater shift if the sequence ended with a discrepant trial (2.26°), compared to when it started with a discrepant trial (1.7°), and the lowest shift was observed when only the fourth event was discrepant (0.9°). This effect of the fourth event was also observed in Fig. 4a, although in smaller magnitude. The effect of each audiovisual trial in the sequence over the auditory shift was not statistically significant in a two-way ANOVA [*F*(4) = 0.00098, *p* = 1].

As discussed above, there is evidence that the subsequent auditory shifts were affected not only by the stimuli in the sequence, but also possibly by longer-term calibration effect. This could explain why all values presented in Fig. 4 are positive. Therefore, these values should not be read as the absolute expected shifts after an isolated sequence of audiovisual events. Regardless, Fig. 4 makes it evident that the most recent audiovisual stimulation directly affects the subsequent auditory localization shift.

Next in this subsection, we provide an analysis to identify what sequential properties are mainly responsible for subsequent perceived auditory shifts. This analysis was solely based on stimulus sequence attributes ‘matching’ or ‘discrepant.’ In a first exploratory stage, many sequence properties were identified and several statistical tests were performed, to identify which stimulus properties could affect the results. The properties originally identified were as follows: whether each of the 5 audiovisual sequence events (SQ1 to SQ5) was or not discrepant; the proportion of discrepant trials in sequence (*D*), ranging from 0 to 1, where 0 was an all-matching sequence and 1 an all-discrepant sequence; the proportion of maximum consecutive discrepant trials in sequence (Max*D*); and the maximum proportion of consecutive discrepant trials at the end of the sequence (MaxDend). Therefore, all tested parameters ranged from 0 to 1, where 1 corresponded to more discrepant events and therefore were expected to correlate positively with the subsequent auditory shift.

To make a pre-selection of predictive properties, several tests were performed to assess whether the properties correlated with the subsequent auditory shifts. The test Shapiro–Wilk did not confirm normality in the data for any of the tested continuous properties. Therefore, Pearson’s tests were applied. Results from this preliminary test are presented in Table 2. All correlation coefficients were quite low, except for the property SQ5. The three properties that had a significant correlation with the auditory shift were SQ5, *D* and Max*D*.

**Table 2:** Pearson’s correlation coefficient and significance between each identified audiovisual sequence property and the subsequent auditory shift

	Pearson’s correlation	Sig. (2-tailed)
SQ1	0.26	0.15
SQ2	0.222	0.223
SQ3	0.208	0.254
SQ4	0.102	0.578
SQ5	0.854**	0.00
*D*	0.33*	0.02
Max*D*	0.269*	0.029
Max*D*end	0.237	0.25

For the next stage of analysis, we selected the attributes with significant correlation with the shift. All sequence events were also included in the prediction tests, since it was hypothesized that including the full stimuli sequence would lead to good predictions of the subsequent effects. This selection process was ultimately subjective, and therefore it must be assumed that other properties might exist that can be as predictive, or more, as the properties selected.

To test all the sequence properties, looking into which ones are more relevant, a weighted parameter sum was computed. It was hypothesized that the auditory shift would be equal to

2 $${\text{SH}}_{\text{A}} = \mathop \sum \limits_{i}^{n} W_{{P_{i} }} P_{i}$$

where $P_{i}$ is each tested parameter and $W_{{P_{i} }}$ is its weight. The weights were computed based on the best-fitting linear weighting of the stimulus properties PA. The best-fitting weighting was derived by assuming a system of linear equations, one for each sequence type. A matrix **P** was created with each sequence type as column vector and each tested parameter set as row vector. For example, when testing only if each event in a sequence was discrepant or not, **P** had only 5 columns, corresponding to each audiovisual trial in the sequence, and each line had a combination of 0 and 1 values, where 0 were matching trials and 1 were discrepant trials, as presented in Eq. (3):

3 $${\mathbf{P}} = \left[ {\begin{array}{*{20}c} {{\text{SQ}}_{1,0} } & \cdots & {{\text{SQ}}_{5,0} } \\ \vdots & \ddots & \vdots \\ {{\text{SQ}}_{1,1} } & \cdots & {{\text{SQ}}_{5,1} } \\ \end{array} } \right]$$

Another matrix **SH** was created with the subsequent auditory shifts per sequence type as column vector. The weights (**W**) are defined as the multiplier of **P** with product **SH**:

4 $${\mathbf{SH}} = {\mathbf{P}} *{\mathbf{W}}$$

The best weighting is found by deriving the pseudo inverse given by

5 $${\mathbf{W}} = \left( {{\mathbf{P}}^{\varvec{T}} *{\mathbf{P}}} \right)^{ - 1} *{\mathbf{P}}^{\varvec{T}} *{\mathbf{SH}}$$

which provides the least squares solution to a system of linear equations and calculates multiple linear regression weights. Then, all predictions were tested by analyzing the linear regressions between predicted and obtained auditory shifts. A summary of all the tested predictions is shown in Table 3.

**Table 3:** In the first column, there are the properties used in each tested **P** matrix; in the second column are the final Spearman coefficients of determination from the correlation between prediction and obtained auditory shift (*r* ^2^); the third column displays the corrected Akaike’s Information Criterion values (AIC_c_), and the right column displays the relative probability of each model best describing the auditory shifts

Properties used in system of equations	*r* ^2^	AIC_C_	AICW_*i*_
SQ1, SQ2, SQ3, SQ4, SQ5	0.844	−79.320	0.143
SQ1, SQ2, SQ3, SQ4, SQ5, *D*	0.855	−72.27	0.004
SQ1, SQ2, SQ3, SQ4, SQ5, Max*D*	0.843	−71.902	0.004
SQ1, SQ2, SQ3, SQ4, SQ5, *D*, Max*D*	0.740	−49.438	0.000
SQ5, *D*	0.855	−82.364	0.655
SQ5, Max*D*	0.855	−47.673	0.000
SQ5, *D*, Max*D*	0.855	−79.926	0.194

We found that all models predicted the auditory shift fairly well. To choose the best-fitting model, we used Akaike’s Information Criterion (AIC) (Akaike 1974; Symonds and Moussali 2011). This method allows for the testing of several predictive models at once. It accounts for a different number of parameters used in each model, and favors parsimony. The lower the AIC value, the better the prediction. AIC values were computed using the residual sum of squares (RSS) obtained from the linear regressions, as given by

6 $${\text{AIC}} = n\left[ {{ \ln }\left( {\frac{\text{RSS}}{n}} \right)} \right] + 2K$$

where *n* is the sample size and *K* is the number of fitted parameters. Here, we only considered the number of properties used in each matrix as *K.* No signs of data overdispersion, which would require additional corrections, were observed. Since *n/K* was inferior to 40, a modified AIC for small sample size (AIC_C_) was calculated:

7 $${\text{AIC}}_{\text{C}} = {\text{AIC}} + \frac{2K(K + 1)}{n - K - 1}$$

Then, we compared each prediction by computing Akaike’s model weights (AICW):

8 $${\text{AICW}}_{i} = \frac{{{ \exp }\left( { - \frac{1}{2}\Delta _{i} } \right)}}{{\mathop \sum \nolimits_{r = 1}^{R} { \exp }\left( { - \frac{1}{2}\Delta _{r} } \right)}}$$

This model comparison is based on Δ_*i*_ calculated as the difference between the best AIC_C_ values and each tested AIC_C_. Akaike’s weights are always values from 0 to 1 and are often read as the percentage by which a model best predicts data. The final values can be read from Table 3. It was found that, with a probability of 66 %, considering only the last trial of the sequence (SQ5) and the overall number of discrepant trials in the sequence (*D*) provided the best prediction. Therefore, the best prediction is calculated as

9 $${\text{SH}}_{\text{A}} = W_{{\text{SQ}5}} {\text{SQ}}5 + W_{\text{D}} D$$

where the property weights were as follows: *W*_SQ5_ = 0.88 and *W*_D_ = 3.42.

## Discussion

This study had two main goals. Firstly, we explored recent evidence suggesting that a single brief exposure to an audiovisual discrepant trial could lead to a subsequent auditory localization shift. In particular, we analyzed whether prior multisensory presentation of auditory-visual stimuli with discrepant positions leads to auditory localization shifts. Secondly, we identified the factors that predict these shifts in auditory localization.

The auditory trials at the end of the audiovisual sequences were on average shifted by 2.2° in the direction of the light source. This finding is in line with that by Wozny and Shams (2011b) where, after exposure to a single trial of audiovisual discrepant stimuli, the subsequent auditory trial had a shift in perceived auditory source position. However, Wozny and Shams also found that the auditory shifts occurred mostly when, in the preceding audiovisual trial, visual and auditory stimuli were perceptually fused, or more specifically perceived as co-localized. Here we find that the mechanism can be independent of such fusion. Indeed, we found that in audiovisual discrepant trials the visual and auditory stimuli were very rarely perceived as co-localized, which is a requirement for multisensory fusion. This has never been clearly analyzed in previous studies. Most studies on the ventriloquism aftereffect exposed subjects to prolonged audiovisual stimulation where visual and auditory stimuli were discrepant in space, but did not ask the subjects to report whether stimuli were co-localized or not (e.g., Lewald 2002; Frissen et al. 2003, 2012). Radeau and Bertelson (1974) obtained an auditory shift after prolonged exposure to audiovisual stimulation. They clearly stated that no subject reported noticing the audiovisual spatial discrepancy. Also, recent models to explain the ventriloquism aftereffect have assumed perceptual fusion (Sato 2007; Wozny and Shams 2011a). In those models, it is assumed that subjects infer the causality of audiovisual stimulation. If visual and auditory stimuli are inferred to be caused by the same event (fused), then an effect over the auditory localization is expected. Otherwise, the auditory estimate is predicted to remain unbiased. Therefore, the finding that we report here calls for a more comprehensive model that accounts for auditory adaptation in the absence of perceptual fusion.

Regarding evidence collected during audiovisual trials, it was found that responses to visual stimuli had no bias toward the auditory sources. Interestingly, this finding contradicts a commonly accepted audiovisual integration assumption that localization under audiovisual stimulation will always have reduced or optimized variance (Alais and Burr 2004). However, it can easily be argued that in audiovisual trials, subjects had a more divided attention, since they were asked to localize two stimuli, while in unimodal trials they only had to focus on a single stimulus. This attentional difference could possibly explain the higher variance we found in audiovisual trials. In discrepant trials, we found a bias in auditory localization toward the light position. Importantly, this bias occurred without perceptual fusion of visual and auditory stimuli. In other words, visual and auditory stimuli were, for the most part, not perceived as co-localized in discrepant trials. This is not a novel finding, as it is known for a long time that attraction of the auditory localization by conflicting inputs occurs even when fusion is not reported (Bertelson and Radeau 1981). This attraction is often called cross-modal bias, as opposed to ventriloquism, which requires perceptual fusion.

Interestingly, in matching trials, visual and auditory trials were not always perceived as co-localized. Indeed, when presented with synchronous light and sound, subjects often perceived the sound as slightly shifted in a small, but marginally significant effect. The effect cannot be attributed to a basic sensory bias toward the visual stimulus, since in that case it was co-localized. It could rather reveal a longer-term sensory recalibration that is triggered by accumulated previous exposure to discrepant audiovisual stimuli.

But there are mixed findings in this study regarding the existence of a long-term recalibration mechanism, running in parallel with the effects of the most recent audiovisual stimulation. We found that time did not seem to affect this recalibration mechanism. It could have been expected that, as the experiment progressed, the auditory shifts would become larger. However, what was obtained was that from the first auditory trial a shift was already observed, and then it did not change significantly. Frissen et al. (2012) also analyzed the effect of time on the auditory recalibration. They found that auditory localization shifts can be obtained quite fast, in 18–24 exposures, but they do increase very rapidly during those first exposures. Here we did not find initial increases in the same magnitude. This does not mean that there was no effect. It can be that subjects were extremely fast to produce an initial adjustment and then kept it constant.

As a second goal in this study, we analyzed the factors that predict the auditory localization shift. All these results are novel.

Analyzing the impact of each audiovisual trial on the subsequent auditory shift, our results show that all five audiovisual trials had an effect, but it was the last trial that led to greater changes. Several sequence properties were identified as correlated with the subsequent auditory shift: the last audiovisual trial, the proportion of discrepant trials in a sequence, and the maximum consecutive discrepant trials. The process of identifying the best candidate properties was exploratory in nature, and therefore, it is possible that other properties would have provided good predictions, too.

In the chosen model, the main features that better explain the auditory shift were indeed the type of audiovisual stimulation in the most recent trial, combined with the overall number of discrepant trials in the sequence. We obtained a good prediction of the auditory shifts by assigning weights to each of these properties.

It is hard to tell whether our prediction would apply to other studies, with different methods. Wozny and Shams (2011b) did not design their experiments specifically to analyze sequential effects. Therefore, in the trials preceding the auditory localization estimates, all types of stimuli could occur, including unisensory visual or auditory trials. In that study, the impact of a discrepant trial could be observed when it happened up to three trials before the auditory judgment. But the impact of the last audiovisual trial was more evident. There was also some evidence that more consecutive discrepant trials led to larger shifts. Therefore, results do not seem to be conflicting, but it is unclear whether our model would apply to their findings. Further studies should be carried out to analyze the robustness of these predictions, namely with larger sequences and with varying discrepant stimulation.

In sum, auditory localization shifts could be observed following short exposure to brief audiovisual discrepant stimulation. For the first time, it is revealed that, within recent sensory experience, all audiovisual stimuli are actually relevant, but that it is the last one that is weighted most.
